# Medical laboratory accreditation in a resource-limited district health centre laboratory, Addis Ababa, Ethiopia

**DOI:** 10.4102/ajlm.v8i1.793

**Published:** 2019-09-19

**Authors:** Daniel M. Desalegn, Boja D. Taddese, Nebiyou Yemanebrhane, Mulye S. Getahun, Kumera T. Kitila, Tariku T. Dinku, Kassahun D. Asferie, Elizabeth A. Wolde, Gemechis B. Tura, Tilahun B. Mersha, Alemayhu W. Rorissa, Daniel D. Wondimagegnehu, Tinsae K. Hailu, Abrham T. Bika

**Affiliations:** 1 Ethiopia Public Health Institute, Addis Ababa, Ethiopia; 2 Addis Ababa Public Health Research and Emergency Management Core Process, Addis Ababa City Administration Health Bureau, Addis Ababa, Ethiopia; 3 Addis Ketema District Health Center Laboratory, Addis Ketema, Addis Ababa, Ethiopia

**Keywords:** Laboratory accreditation, ISO 15189, World Health Organization African Region, stepwise laboratory accreditation preparedness scheme

## Abstract

**Background:**

Improving the quality of medical laboratory services is a high priority in many countries. However, quality management systems for laboratories in resource-limited settings are often inadequate.

**Objectives:**

This article shares the experiences, benefits and challenges of the laboratory journey towards accreditation in a primary healthcare laboratory in Addis Ababa, Ethiopia.

**Methods:**

A retrospective review of laboratory records in Addis Ketema Health Center was conducted from 2012 to 2015. The study was supplemented by observations from some of the authors of this article who worked in the laboratory.

**Results:**

The laboratory journey towards accreditation began with a baseline assessment in 2012 using the World Health Organization African Region Stepwise Laboratory Quality Improvement Process Towards Accreditation; the baseline score was 78 points (0 stars). After mentorship support, the laboratory improved to 198 points (3 stars) in 2013 and 249 points (5 stars) in 2014. The laboratory scaled up to International Organization for Standardization 15189 requirements and received limited-scope accreditation for tuberculosis sputum microscopy and hematology tests in 2015. After adopting and implementing the standards, steady improvement was observed in the reliability of the laboratory services. Lack of resources was the major challenge the laboratory encountered.

**Conclusion:**

Even though a remarkable quality performance improvement was observed over the entire process, inadequate skilled personnel was the major challenge identified in the road towards accreditation. Therefore, an appropriate, workload-based staffing structure should be developed to improve and sustain medical laboratory quality standards in resource-limited settings.

## Background

In developing countries, the expansion of laboratory diagnostic infrastructure has increased to the extent of meeting the needs of evidence-based treatment.^1^ Along with investing in accessibility, simultaneous improvements in the quality of laboratory services are needed to ensure the quality of health services.^1,2^ Laboratory service in resource-limited settings is very weak and quality assurance system practices are either very weak or do not exist at all.^3^ Laboratory testing errors can mislead clinicians and cause serious harm to patients, but the errors can be reduced by establishing a comprehensive laboratory quality management system.^1,4^

The implementation of a laboratory quality management system enables the healthcare system to provide reliable services and strengthens the overall quality of patient care. Moreover, it yields long-term benefits in the quality of healthcare, optimises the expenditure of healthcare resources as well as supporting health policy.^5^ Poor infrastructure, low human resource capacity and inappropriate technologies are notable challenges to the implementation of clinical laboratory international quality standards in resource-limited regions. To address this challenge, the World Health Organization African Region (WHO-AFRO) established a stepwise approach in developing countries.^6,7^ Laboratories that demonstrate outstanding performance in the WHO-AFRO process are accelerated toward International Organization for Standardization (ISO) 15189 clinical laboratory accreditation.^6,8^

In many developed countries, accreditation of medical laboratories has been established for several decades.^9^ However, few laboratories have been accredited to date in Ethiopia. The WHO-AFRO Stepwise Laboratory Quality Improvement Process Towards Accreditation (SLIPTA) was launched in Ethiopia in 2009 in order to strengthen the quality of laboratory services.^10^ Laboratories in the public health system of Ethiopia are divided into four tiers: district/health centre laboratories, hospital laboratories, regional reference laboratories and national reference laboratories.^10^ The Addis Ketema District Health Center Laboratory is one of the public health facilities under the Addis Ababa City Administration Health Bureau. The laboratory has implemented the SLIPTA standards, and its performance improved from a baseline of 0 stars in 2012 to 5 stars in 2015. This outstanding performance on the WHO-AFRO checklist encouraged the health centre management and laboratory staff to apply for accreditation to ISO 15189:2012, a standard with particular requirements for quality and competence for medical laboratories. This article shares the experiences, benefits and challenges of implementing the WHO-AFRO SLIPTA process and ISO 15189 clinical laboratory accreditation in a primary healthcare laboratory in Addis Ababa, Ethiopia.

## Methods

### Study setting and context

A retrospective review of existing data for WHO-AFRO SLIPTA external audit reports and quality indicators at Addis Ketema District Health Center Laboratory from 2012 to 2015 was conducted. The health centre was established in 1963 under the Addis Ababa City Administration Health Bureau. In Addis Ababa, healthcare facility expansion has improved physical access to health services with an emphasis on primary healthcare units, resulting in the potential for an estimated 100% health service coverage of the city.^11^ The city has 47 hospitals, 573 private clinics (204 higher-, 226 medium-, 143 lower-level clinics) and 100 government health centres.^12,13^ Regarding clinical laboratory accreditation practices at the time of this assessment, only four hospitals, two regional laboratories and the Addis Ketema District Health Center Laboratory had received limited-scope accreditation to the ISO 15189 laboratory standards.

The study was supplemented by the observation and experience of some of the authors of this article who worked in the laboratory as experts or managers throughout the accreditation process. Data on the laboratory quality improvement process and key laboratory quality indicators, including the WHO-AFRO SLIPTA external audit findings, were collected by using a data extraction form. The WHO-AFRO external audit reports were assessed on the basis of the SLIPTA checklist scoring system which weighted marks out of a total of 258 points. The checklist star rating was as follows: 0–142 points: 0 stars, 143–165 points: 1 star, 166–191points: 2 stars, 192–217 points: 3 stars, 218–243 points: 4 stars and 244–258 points: 5 stars.^14^ All collected data were checked for completeness, transcribed, coded, categorised and analyzed to indicate the impact of implementing quality standards on the laboratory services improvements.

### Operational definitions

#### District health centre

A district health centre is a health facility that functions as a basic first-line unit and that provides primary healthcare services.

#### Quality indicators

A quality indicator is an objective measure that potentially evaluates all performance improvements in laboratory services based on ISO 15189 standards (turn-around time, external laboratory assessment, customer satisfaction, laboratory service interruption, equipment downtime, specimen rejection and laboratory supplies stock level).

## Results and discussion

### The road toward laboratory accreditation

The laboratory journey towards accreditation began with a baseline assessment in 2012 using the WHO-AFRO SLIPTA checklist; the baseline audit result was 78 points (0 stars). Major gaps identified at baseline were poor laboratory infrastructure, inconsistent electric power supply, lack of laboratory supplies and knowledge gaps on the standards. In addition, the human power at the laboratory was insufficient to handle the additional workload that resulted from the implementation of the quality management system. This additional work, such as extensive paperwork for adopting laboratory policies, standard operating procedures, manuals, guidelines and other daily task records, was not considered part of the ‘daily tasks’ used to determine the number of laboratory professionals assigned to the health centre.

The laboratory staff and management discussed the gaps identified at the baseline and developed a comprehensive and goal-oriented action plan. The laboratory organised sensitisation meetings and mobilised resources from government and partners for renovation, equipment servicing, laboratory supplies and external quality assessment (EQA) materials. The laboratory rooms were renovated and a backup power supply (generator) was purchased using funds obtained from the Addis Ketema sub-city health offices. For the implementation of a laboratory information system, computers were donated by Technical Support for the Ethiopia HIV/AIDS Initiative, which is an international non-governmental organization at the Johns Hopkins Bloomberg School of Public Health, Johns Hopkins University, Baltimore, Maryland, United States.

**FIGURE 1:**
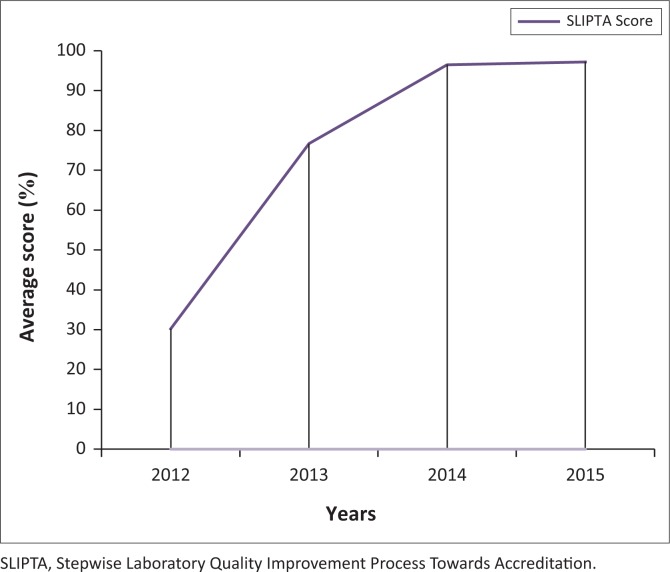
WHO-AFRO SLIPTA score trend at Addis Ketema District Heath Center Laboratory, 2012 – 2015.

Even though an appropriate, workload-based staffing structure was not developed in Ethiopia to implement the laboratory quality standards at the health centre level, the health centre’s management hired two additional laboratory professionals after getting permission from the civil services agency of Addis Ababa to minimise the workload on laboratory staff. Training on the standards and laboratory mentoring services was requested from the Addis Ababa City Administration Health Bureau, the Ethiopian Public Health Institute and other partners. In addition, the German Society for International Collaboration covered the fee for EQA materials. After a year, the laboratory was enrolled in a national EQA scheme, which is one of the criteria for laboratory accreditation. After one year of mentorship support, the laboratory improved from 78 points (0 stars) on the SLIPTA audit in 2012, to 198 points (3 stars) in 2013, 249 points (5 stars) in 2014 and 251 points (5 stars) in 2015 (Figure 1).

This outstanding performance of the laboratory on the WHO-AFRO checklist encouraged us to seek international accreditation. Preparation for accreditation required the involvement and commitment of the entire staff, organisation and partners. We began requesting consultation services from the German Society for International Collaboration, one of the international organisations working in Ethiopia, and the Addis Ababa City Administration Health Bureau. They provided consultancy services and coaching on how to implement ISO 15189 accreditation requirements.

The full scope of clinical laboratory accreditation was difficult to implement in our setting due to limited resources and inadequate experience. Therefore, based on the availability of internal quality control materials, EQA schemes and other resources in the study setting, the laboratory management and the consultancy organisation selected tuberculosis direct sputum smear microscopy and hematology tests for accreditation.

The organisational structure of the laboratory started with the laboratory head, who directly reports to the health centre management, followed by the quality officer, supervisor and ends with the signatory of each section. The laboratory management worked to ensure the responsibility, authority and interrelationships as they were defined and documented in the quality manual. We established different teams and gave them the responsibility for adopting the standard. Different teams coordinated on different activities in the areas that needed improvement. The document and record team developed and adopted various documents, including the manual, job-aids and technical and managerial standard operating procedures. The laboratory management customised the laboratory quality policy, staff competency assessment guideline and incidence reporting format. Other teams and personnel were assigned as required for the laboratory quality standard, including an internal audit team, customer officer, quality officer, safety officer, and logistics officer.

All levels of the laboratory management were tasked with ensuring continued workflow and communication among the staff, so that complaints could be quickly identified, addressed and resolved. The laboratory staff attended regular weekly meetings to assess progress in every unit and any challenges encountered. In addition, the laboratory had monthly meetings with the health facility’s upper management and clinicians, which facilitated an open forum for presenting problems and opportunities for improvement.

In addition, a computer-based laboratory information system was adapted locally. It serves as the interface between laboratory testing services and out-patient department services. The system receives laboratory requests from out-patient departments, releases results to clinicians and is used to monitor all laboratory quality indicators. Moreover, in order to take corrective action, the laboratory information system notifies the laboratory of any deviation from predefined quality standards, including panic results and unreleased test results within the defined turn-around time.

To check the sustainability of the implementation of the systems, the laboratory routinely monitored all quality indicators, including turn-around time, sample rejection rate, daily quality control, refrigerator and room temperature monitoring, supply management, equipment performance and internal and external complaints. External quality assessment performance, customer surveys and safety audit findings were evaluated quarterly. Personnel competency assessment and internal audit findings were reviewed twice a year. The monitoring process helped to regulate the entire laboratory process from the pre-analytical to the post-analytical phases.

The laboratory management established different agreements with other laboratories in the region for inter-laboratory comparison, referral and backup services to ensure quality services and reduce service interruptions. Following all these preparations, the laboratory requested ISO 15189 certification from the Ethiopian National Accreditation Offices. On 19 November 2015, 3 years after commencing the journey, the laboratory received limited-scope accreditation for tuberculosis direct sputum smear microscopy and hematology tests^15^ and became the first ISO 15189-accredited district health centre laboratory in Ethiopia. The lesson learned from this partial clinical laboratory accreditation may encourage the organisation to scale up to the full scope of accreditation.

### Challenges on the road toward accreditation

Lack of internal quality control materials and other reagents, inconsistent electric power supply and a limited number of trained laboratory personnel were the major challenges the laboratory encountered and had to overcome. In addition, equipment service and calibration were challenging to procure at minimal cost in the study setting. This problem was due to the limited availability of qualified engineers, high maintenance costs and lack of equipment and spare parts. This led to extended equipment downtime, service interruptions and delays in service delivery, which resulted in increased complaints from clinicians and customers. Lack of funds for renovation of existing structures, inadequate laboratory rooms and unsuitable laboratory design were additional challenges in the accreditation process.

To implement the standards, we performed major alterations to the daily laboratory operation, which created an additional workload and required resources; owing to these problems, some staff and management members did not accept the standard at the beginning. However, regular training, the inclusion of additional staff, partners’ support and pertinent discussion on the benefits of the standards led to increased understanding and cooperation.

In summary, continual training encouraged members of staff to read about the standards and share experiences with quality management. Moreover, supplies and equipment service agreements with local vendors, government and partners’ support, leadership and staff commitment were crucial to overcome the challenges.

### Accreditation benefits

**FIGURE 2:**
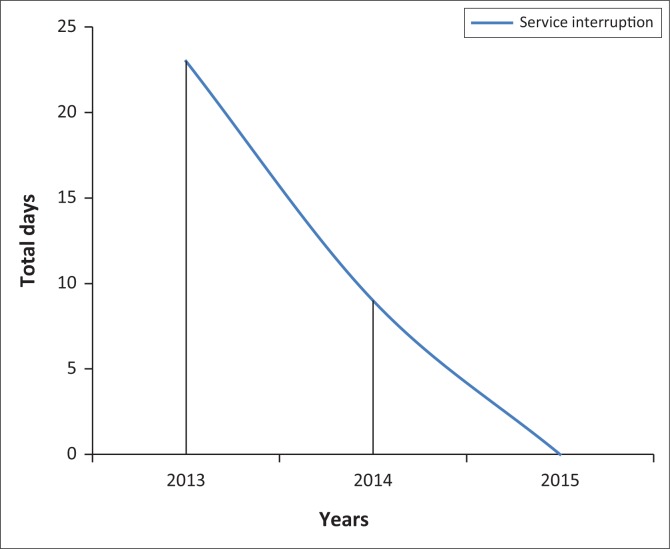
Trends in laboratory service interruption, Addis Ababa, Ethiopia, 2013 – 2015.

On adopting the standards, steady improvement was observed in the reliability, reproducibility, traceability and uniformity of laboratory services. We established effective inventory management systems that enabled us to forecast laboratory supplies, reduce reagent wastage and service interruptions due to stock-out. As a result, for 18 consecutive months from March 2014 to August 2015, there were no interruptions to tuberculosis direct sputum smear microscopy and hematology testing services. Overall, laboratory diagnostic service interruptions significantly decreased from 2013 to 2015 (Figure 2).

Implementation of the standards aided profound improvement in EQA performance. Tuberculosis direct sputum smear microscopy slide test performance (blinded, rechecking) improved from 97.9% to 100% and hematology proficiency improved from 84.2% to 100% (Table 1). These improvements were due to increased staff competence, which enabled the laboratory to establish a reliable system that provides accurate, reliable and quality service delivery.

**TABLE 1:** Tuberculosis direct sputum smear microscopy external quality assessment and hematology proficiency test performance, Addis Ababa, 2013 - 2015.

Year	Direct sputum smear microscopy slide (blinded, rechecking)						Hematology proficiency testing: Performance (%)
	False positive results		False negative results		Performance		
	No.	%	No.	%	No.	%	
2013	1/27	3.7	3/171	1.8	194/198	97.9	84.2
2014	-	0.0	1/187	0.5	186/187	99.5	100.0
2015	-	0.0	-	0.0	173/173	100.0	100.0

The laboratory scheduled the assessment of quality indicators which helped in timely identification of system weaknesses and rapid resolution of identified problems. It assisted in rapid detection of ineffective systems and led to taking corrective actions. The laboratory quality indicator results improved after 2013. On average turn-around time declined from 6 h to 55 min for hematology tests and 3 days to 1 day for tuberculosis direct sputum smear microscopy tests. Mean equipment downtime declined from one month to one and a half days, stock-out of supplies declined from 11 days to zero days. Higher compliance with sample collection guidelines and sample acceptance and rejection criteria were accompanied by a reduction of sample rejection rates from 3.7% in 2013, to 2.1% in 2014 and 0.43% in 2015. The scheduled assessment of quality indicators benefited the laboratory in tracing errors and complaints. Consequently, overall customer satisfaction for all services increased from 63% in 2013 to 87% in 2015 (Table 2).

**TABLE 2:** Laboratory quality indicators performance, Addis Ababa, Ethiopia, 2013 - 2015.

Quality indicators	2013	2014	2015
Customer satisfaction (%)	63.0	84.0	87.0
Total days of laboratory service interruption	21.0	11.0	0.0
Total days of equipment downtime	33.0	5.0	1.5
Specimen rejection (%)	3.7	2.1	0.43
Test results delivered within defined turn-around time (%)	68.0	96.1	96.8
Total days of supply stock-outs	11.0	4.0	0.0

### Conclusion

The lesson learned from our experience is that clinical laboratory accreditation in a resource-limited primary healthcare laboratory is achievable when the system is implemented using a stepwise approach, such as scaling up from WHO-AFRO SLIPTA to a partial-scope accreditation, and scaling up to the full scope of accreditation. However, limited resources and inadequately skilled laboratory personnel were the major challenges identified in our journey towards accreditation. Therefore, considering the quality standards, an appropriate, workload-based staffing structure and resources, as well as the well-coordinated commitment of the entire staff, organisation, mentors and stakeholders, must be sought to adopt and sustain medical laboratory quality standards in a resource-limited setting.

Lessons learned

- The right strategy, teamwork and leadership are crucial for the implementation of a laboratory quality management system.
- Clinical laboratory accreditation creates opportunities to improve the quality of healthcare services through provision of accurate, reliable and timely laboratory test results to customers.
- An appropriate, workload-based staffing structure, positive staff attitude towards accreditation, as well as the well-coordinated commitment of the entire staff, must be sought when adopting and sustaining medical laboratory accreditation in resource-limited settings.
